# Development and validation of a targeted next generation DNA sequencing panel outperforming whole exome sequencing for the identification of clinically relevant genetic variants

**DOI:** 10.18632/oncotarget.22116

**Published:** 2017-10-26

**Authors:** Eirwen M. Miller, Nicole E. Patterson, Jenna Marcus Zechmeister, Michal Bejerano-Sagie, Maria Delio, Kunjan Patel, Nivedita Ravi, Wilber Quispe-Tintaya, Alexander Maslov, Nichelle Simmons, Maria Castaldi, Jan Vijg, Rouzan G. Karabakhtsian, John M. Greally, Dennis Y.S. Kuo, Cristina Montagna

**Affiliations:** ^1^ Department of Genetics, Albert Einstein College of Medicine, Bronx, NY 10461, USA; ^2^ Department of Obstetrics & Gynecology and Women's Health, Division of Gynecologic Oncology, Montefiore Medical Center, Bronx, NY 10461, USA; ^3^ Department of Pathology, Montefiore Medical Center, Bronx, NY 10461, USA

**Keywords:** target sequencing, next generation sequencing, endometrial carcinoma, tumor recurrence

## Abstract

Next generation sequencing (NGS) technologies have revolutionized our approach to genomic research. The use of whole genome sequencing (WGS), whole exome sequencing (WES), transcriptome profiling, and targeted DNA sequencing has exponentially improved our understanding of the human genome and the genetic complexities underlying malignancy. Yet, WGS and WES clinical applications remain limited due to high costs and the large volume of data generated. When utilized to address biological questions in basic science studies, targeted sequencing panels have proven extremely valuable due to reduced costs and higher sequencing depth. However, the routine application of targeted sequencing to the clinical setting is limited to a few cancer subtypes. Some highly aggressive tumor types, like type 2 endometrial cancer (EC), could greatly benefit from routine genomic analysis using targeted sequencing. To explore the potential utility of a mid size panel (~150 genes) in the clinical setting, we developed and validated a custom panel against WGS, WES, and another commercially available targeted panel. Our results indicate that a mid size custom designed panel is as efficient as WGS and WES in mapping variants of biological and clinical relevance, rendering higher coverage, at a lower cost, with fewer variants of uncertain significance. Because of the much higher sequencing depth that could be achieved, our results demonstrate that targeted sequencing outperformed WGS and WES in the mapping of pathogenic variants in a breast cancer case, as well as a case of mixed serous and high-grade endometrioid EC, the most aggressive EC subtype.

## INTRODUCTION

WGS and WES have emerged as valuable tools for the identification of driver mutations in a variety of tumor types, as well as for the identification of actionable mutations [1–3]. Though targeted therapies have shown promise in chronic myeloid leukemia, lung cancer, and melanoma, actionable mutations are currently limited to a few dozen genes due to the small number of compounds on the market that are mutation specific [4–7]. As a consequence, when searching for a few specific targetable mutations, the sheer volume of data generated by WGS or WES proves burdensome from standpoints of both economy and labor [8–9]. The expense combined with the time needed for sequencing and data analysis excludes WGS and WES as efficient means of variant identification in the clinical setting [10].

We custom designed an in house panel (the Einstein Custom Cancer Panel, ECCP) to investigate specific malignancies of interest at our institution. The goal of our study was to validate our panel against WGS, WES, and another commercially available targeted panel and explore its potential clinical application. The panel we designed and validated in this study differs from the design commonly used in targeted sequencing profiling because of the larger size (~150 genes compared with a dozen commonly used in clinical settings). This offers the advantage of being suited to the analysis of a variety of cancer subtypes, even those with a more unique genomic profile.

As a proof of principle, we explored the potential of the ECCP to identify clinically relevant mutations in one breast cancer case, as well as an uncommon but highly aggressive endometrial cancer (EC) subtype. Recurrent type 2 EC tumors are poorly responsive to current cytotoxic chemotherapy options [11,12]. Identifying targetable pathogenic somatic variants in type 2 EC patients may provide opportunities to treat women with targeted therapies when curative potential exists, i.e., in the adjuvant rather than recurrent setting.

Our results reveal that a mid size (~150 genes) targeted sequencing panel is as efficient as WGS in mapping variants of biological and clinical relevance, providing higher coverage at a lower cost. We found high sequencing coverage to be an asset in our analysis. Tumors are typically sequenced to depths of 75x–100x (WES) or 30x–50x (WGS), which may be inadequate to analyze tumors that are aneuploid, clonally heterogenous, or impure due to the contamination of non tumor cells [13]. The latter may endanger inaccurate diagnosis, especially in the setting of a diagnostic biopsy where the ratio of tumor to non-tumor cells may be unknown. As suggested for other sequencing-based diagnostic tests, high coverage increased sensitivity and allowed the identification of a tumor driver *TP53* mutation missed by whole exome analysis.

Collectively, our results suggest that targeted sequencing represents an unrecognized and powerful tool to support therapy decisions, especially for aggressive, less analyzed tumor types.

## RESULTS

### Design of the Einstein Custom Cancer Panel (ECCP)

To investigate somatic genomic alterations common to a variety of solid tumors, including breast and gynecologic malignancies, we developed the Einstein Custom Cancer Panel (ECCP), a custom cancer-focused targeted gene panel. On the basis of an extensive literature review, 156 highly cited and frequently mutated oncogenes and tumor suppressor genes (see Supplementary Appendix 1 for a detailed list of included genes) were strategically selected for inclusion in the panel design based on their molecular pathways and their mutation frequency in solid tumors as assessed by mining TCGA through the cBioPortal [14,15] and the Catalogue Of Somatic Mutations In Cancer (COSMIC) [16]. This pathway-based custom panel profiles the mutation spectrum in tumorigenic genes and drug targets along with signaling cascades, DNA repair genes, and growth factor genes. The ECCP was designed for the analysis of small amplicons (~75 bp) using the Ion AmpliSeq technology and is suitable for sequencing using the Ion Proton Sequencer. In total, the design includes 5,610 amplification primers, divided into two primer pools, to cover coding exons of each of the 156 genes.

### Clinical description of patient samples

Samples from two patients, P65 and DL, were used to assess the performance of the ECCP.

P65 is an 82-year-old African American woman with a history of obesity, pulmonary hypertension, coronary artery disease, and congestive heart failure who presented with a right breast mass and breast imaging highly suspicious for breast carcinoma. A core needle biopsy revealed infiltrating poorly differentiated ductal carcinoma. She underwent right mastectomy and axillary nodal dissection for a 3 cm infiltrating high-grade ductal carcinoma with squamous differentiation and lobular carcinoma *in situ* with pagetoid spread into the ducts (pT2N0, ER/PR positive, HER2/neu negative). She received adjuvant endocrine therapy with an aromatase inhibitor (anastrozole) and is deceased secondary to congestive heart failure.

**Figure 1:**
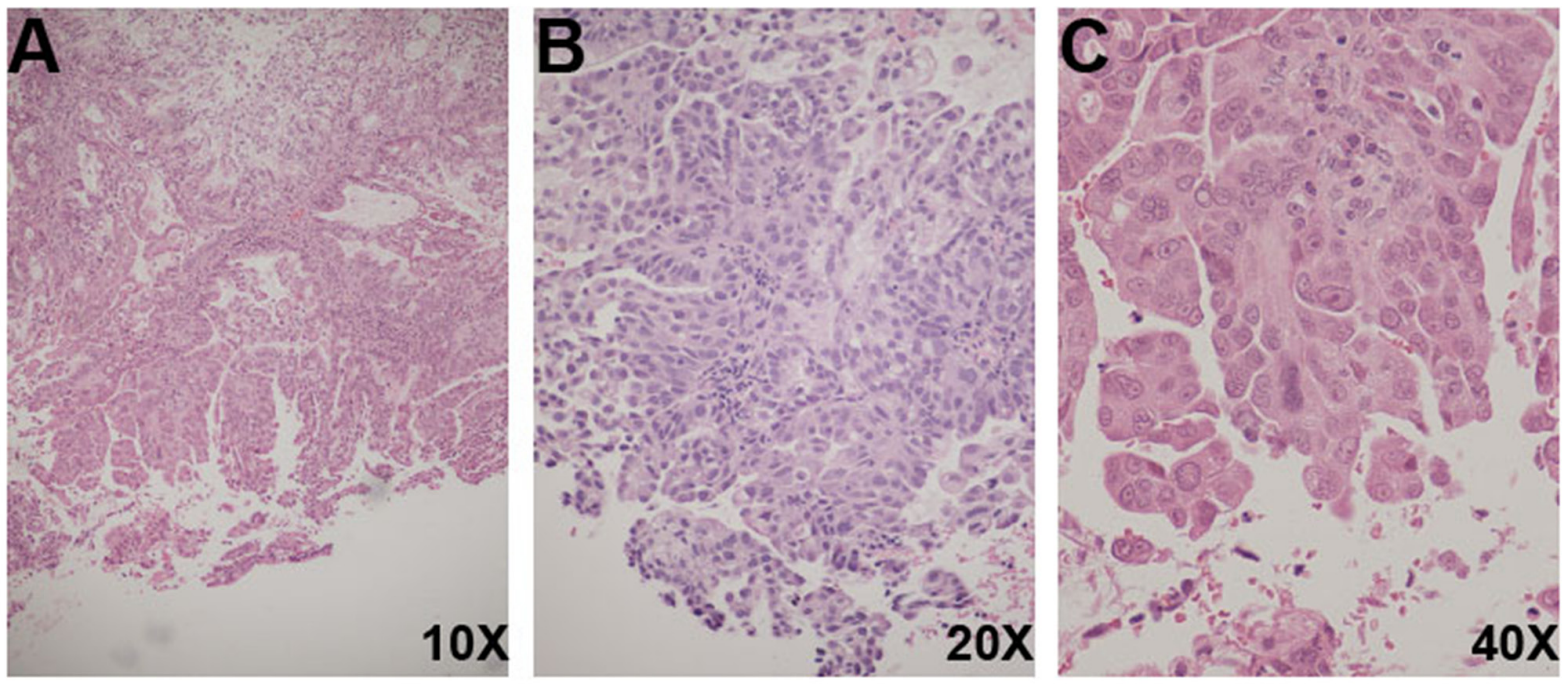
Histological features of DL biopsies **(A)** DL1 (H&E, 10X) Uterine serous carcinoma, papillary projections and pseudoglandular formations. **(B)** DL2 (H&E, 20X) Uterine serous carcinoma, tumor cells with high nucleus-to-cytoplasmic ratio, and mitotic figures. **(C)** DL2 (H&E, 40X) Uterine serous carcinoma, tumor cells with hobnail pleomorphic nuclei, eosinophilic cytoplasm, and prominent nucleoli.

DL is a 44-year-old female who presented with generalized abdominal pain and was found to have a 6 cm uterine mass, ascites, and carcinomatosis on CT scan. She underwent a diagnostic endometrial biopsy (DL1), which demonstrated adenocarcinoma with serous and endometrioid components (Figure 1A) followed by a total abdominal hysterectomy (DL2) with tumor debulking 1 month later. Grossly, the tumor measured 2.5 cm, invading less than 5% of the myometrium. Histopathologic review of the surgical specimen demonstrated stage IVB high grade serous endometrial adenocarcinoma with a minor high-grade endometrioid component (Figures 1B–1C). Immunohistochemically, DL2 was positive for ER, PR, and TP53 and negative for HER2/neu. Commercial Colaris and Integrated BRCAnalysis testing (Myriad Genetics; Salt Lake City, UT) indicated no mutations found in *EPCAM, MLH1, MSH2, MSH6, PMS2, BRCA1,* or *BRCA2* genes. She received adjuvant carboplatin and paclitaxel but her cancer recurred and she underwent a radiographically guided biopsy of an inguinal lymph node metastasis (DL3). She was treated for recurrent disease but ultimately deceased secondary to progressive malignancy.

### Validation of the Einstein Custom Cancer Panel (ECCP) on tissue from patient P65: breast carcinoma

We performed a systematic comparison of data output for tumor (T65) and normal (N65) samples from a variety of sequencing methods: Illumina WES (Illumina, San Diego, CA), and Ion AmpliSeq targeted panels (Thermo Fisher Scientific, Waltham, MA), namely, the company's off-the-shelf Comprehensive Cancer Panel (CCP) and our custom designed ECCP (Table 1). Comparing the data from these sequencing techniques, we sought to assess the extent to which the ECCP may fail to identify mutations predicted to be damaging by in silico analysis, due to the limited number of targets included in the design.

**Table 1:** Specimens for comprehensive sequencing analysis

Sample	Sample Origin	Sample Type	Sequencing Analysis
**N65**	Breast cancer adjacent non tumor tissue	Non-tumor - Fresh	WES Targeted CCP Targeted ECCP
**T65**	Breast cancer tumor	Tumor - Fresh	WES Targeted CCP Targeted ECCP
**DL1**	Endometrial biopsy 12/26/2012	Tumor - FFPE	Targeted ECCP
**DL2**	Hysterectomy specimen 1/2/2013	Tumor - FFPE	Targeted ECCP
**DL3**	Inguinal lymph node (recurrence) 4/17/2014	Tumor - Fresh Frozen	Targeted ECCP WGS (NYGC) RNA-Seq (NYGC)
**DLWB**	Whole Blood	Whole blood	Targeted ECCP WGS (NYGC)

We first compared the variant calling analyses of WES to that of the targeted panels (CCP and ECCP). WES sequencing results were analyzed using VarScan, CCP results with both VarScan and Ion Reporter, and ECCP results with Ion Reporter. The use of different variant callers, with exactly the same filtering parameters, was intentional. VarScan, a high performance variant caller [17], is commonly used in analytical pipelines for the analysis of the cancer genome [18] and therefore, was considered our gold standard. VarScan results were directly compared with Ion Reporter, the analytical pipeline specifically designed for variant calling of data generated by Ion sequencing platforms to allow for a streamlined workflow. We sought to determine whether utilization of this workflow was adequate for identification of pathogenic somatic variants. Variant calls were subject to the same filtering parameters, eliminating non-exonic (UTR), synonymous, and common variants (>1% MAF from the 1,000 genome project, the exome sequencing project, and the Exome Aggregation Consortium), as well as variants with benign impact (PolyPhen-2 score = benign and SIFT <0.05) to identify variants of interest. The variants identified by the analyses performed using Ion Reporter were also subject to visual inspection using IGV.

**Figure 2:**
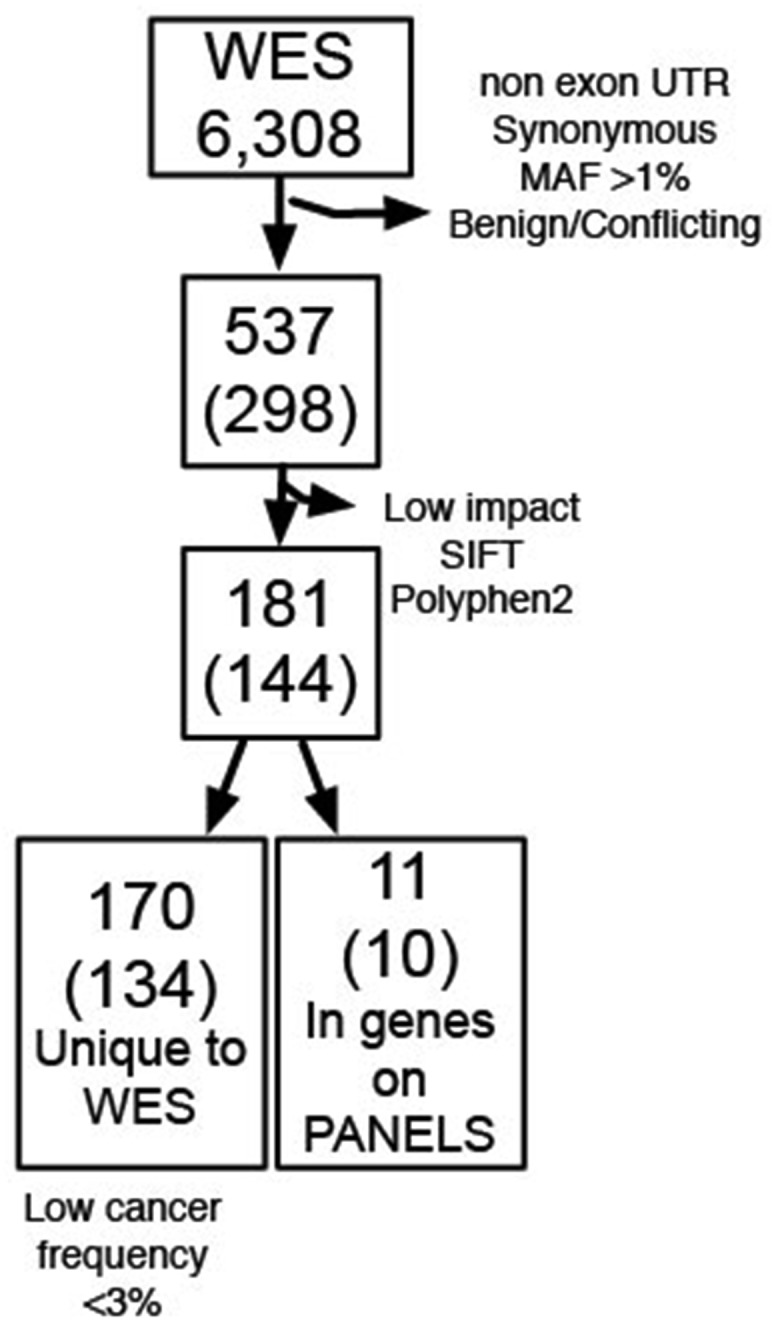
Summary of variants identified in P65 The total number of variants identified by WES (top box) has been subjected to a series of stepwise filtering as indicated on the right side arrows. The number of genes corresponding to the mapping sites of the identified variants is indicated in parentheses. The bottom boxes indicate the number of variants (and genes) missed by targeted sequencing (left) and found in the targeted sequencing panels as well as WES (right).

From the WES raw data, VarScan identified 6,308 somatic variants (Figure 2, Suppllementary Appendix 2) present in T65 but not peripheral blood DNA. After application of the above stated filtering parameters, 537 variants of interest (469 exonic and 68 exon/splicing), mapping to 298 genes, were retained in the dataset (Figure 2). To evaluate whether limiting our analysis to genes included in the targeted panels resulted in missed pathogenic variants, these 537 variants were further filtered to eliminate variants with SIFT and PolyPhen-2 scores predicted to have low impact functional consequences and variants with no or unknown computational scores. The remaining 181 variants with computational scores predicted to be deleterious mapped to 144 genes. 170 of these variants mapped to 134 genes not on the targeted panels, but no mutations in these genes have been reported to occur with greater than 3.25% frequency in all cancers included in the COSMIC database. Furthermore, closer investigation of these genes in relation to all cancers in TCGA indicates that all variants reported therein are putative passenger mutations. Therefore, the genes in which these 170 variants occur are likely not of clinical interest. The remaining 11 variants mapped to ten genes that are on at least one of the targeted panels. Nine of these variants were also identified by targeted panel sequencing. The two remaining missense variants are found in Nuclear Receptor Corepressor 1 (*NCOR1*) and Histone-Lysine N-Methyltransferase (*KMT2C*, previously known as *MLL*3). The *NCOR1* gene is included only in the ECCP, therefore, we do not expect the variant to be called in the CCP analysis. We visually inspected the *NCOR1* variant in IGV for WES and ECCP and found the quality of the WES call to be questionable, given several other nearby modifications occurring with the same frequency as the *NCOR1* variant. Additional database interrogation revealed that the updated MAF of this *NCOR1* variant is 43%, according to the Exome Aggregation Consortium and is reported in only 0.07% of all cancers in COSMIC. The *KMT2C* variant, encoding for an tyrosine to histidine change (p.Y987H), was missed by the targeted panels due to low coverage and poor mapping quality. One additional *KMT2C* (p.G838S) variant was identified in both the WES results and the ECCP results. This variant, which we later determined had an exclusionary MAF, also had poor mapping quality.

**Figure 3:**
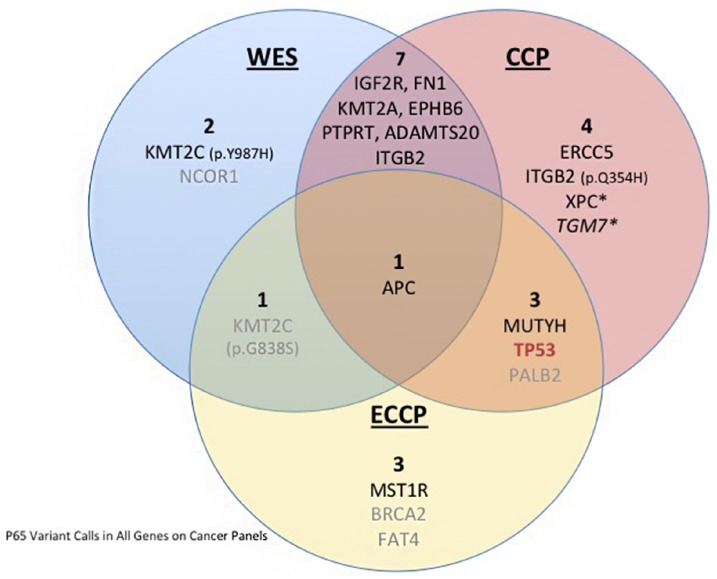
Venn diagram depicting the variants identified in P65 in WES and the targeted CCP and ECCP The blue left circle contains the variants identified by WES; the orange circle contains the variants identified by CCP sequencing, and the yellow circle contains the variants identified by ECCP. The overlapping areas between the circles indicate the common variants, in the center is depicted the APC variant common to all analyses. The star indicates variants classified as possibly pathogenic, in grey we highlight the variants filtered out for lack of evidence support, in italic we indicate the variants the require technical validation. In red we highlight the TP53 driver mutation missed by WES.

To evaluate the performance of Ion Reporter as a variant caller, the raw sequencing data of the CCP (an off-the-shelf AmpliSeq pre-designed targeted sequencing panel covering 409 cancer genes; Supplementary Appendix 3) was analyzed using both VarScan and Ion Reporter. All of the variants called in VarScan were also identified in Ion Reporter and all the variants identified by both VarScan and Ion Reporter, except the MutY DNA Glycosylase (*MUTYH*) variant and XPC Complex Subunit, DNA Damage Recognition And Repair Factor (*XPC*) variants, were also identified by WES. Interestingly, though not called in the WES results, the *MUTYH* variant was identified in the ECCP results. In addition to the variants identified by both variant callers, Ion Reporter identified an additional 8 variants, 4 of which were also identified by WES (Figure 3 and Supplementary Table 1). Of particular interest, the analyses of both the CCP and ECCP sequencing results using Ion Reporter identified a nonsense *TP53* mutation that is classified in ClinVar as pathogenic in relation to Li-Fraumeni syndrome and/or hereditary cancer-predisposing syndrome (Figure 3, in red). This variant, which we validated by Sanger sequencing (Supplementary Figure 1), was not identified by the VarScan analyses of WES and CCP.

Using the WES variant calls as a gold standard, we next assessed the performance of two Ion AmpliSeq targeted sequencing panels: the CCP and the ECCP. The targeted panels have an 18.2% overlap in gene content (87 genes in common out of 478 genes covered in total by the two panels) (Supplementary Figure 2 and Supplementary Appendix 3). The Ion Reporter analysis of the CCP sequencing data identified up to 15 variants meeting our criteria as variants of interest (one variant would require further validation, see below), of which 11 mapped to 10 genes not included in the ECCP. Of these 11 variants, 7 variants (including the 4 mentioned above) were also called in the WES analysis. We found that more inclusive filtering parameters, with less stringent criteria for inclusion based on SIFT and PolyPhen-2, increased the number of variants identified in both the CCP analysis and WES analysis to nine. Allowing for variants with “possibly pathogenic” computational scores to be included, we found that the *XPC* variant and a variant in Transglutaminase 7 (*TGM7*) were now also identified in both the CCP and WES (Figure 3, starred). Visual inspection of the *TGM7* variant in IGV reveals nothing remarkable enough to dismiss the variant, but with only 17 reads on the CCP at that site, we lack the confidence to definitively call it a variant and Sanger validation would be required to confirm this call (Figure 3, italics).

The Ion Reporter analysis of the ECCP initially identified 8 variants, mapping to 8 genes, meeting our criteria as variants of interest. These variants included the *KMT2C* (p.G838S) variant also identified by WES and later filtered out (Figure 3, in grey) and 3 unique variants mapping to genes not included in the CCP (macrophage stimulating 1 receptor-*MST1R*, *BRCA2*, and FAT atypical cadherin 4-*FAT4*).

There are 87 genes in common between the ECCP and CCP, accounting for 21% of the 409 CCP genes. The Ion Reporter analyses of these two targeted panels identified 4 variants common between the two panels, including the validated *TP53* nonsense variant (Supplementary Figure 1). The remaining 11 variants identified by the CCP were in genes not on the ECCP. Likewise, three variants identified by the ECCP but not the CCP were in genes not included in the CCP. One variant, mapping to Adenomatous Polyposis Coli (*APC*), was called by all three analyses. The *BRCA2* and *FAT4* variants identified by the ECCP analysis were later filtered out due to information in ClinVar indicating a lack of pathogenicity, as was the *PALB2* variant identified in the CCP and ECCP analyses (Figure 3, in gray).

The summary of the SNVs and their identification with each sequencing approach is shown in Figure 3 and Supplementary Table 1. Taken collectively, we concluded from these results that the targeted panels adequately identified the clinically relevant mutations in P65.

As a final test for the ECCP, we performed sensitivity and specificity calculations by examining all variants (including non-coding and synonymous variants) called by both the WES and ECCP analyses. Based on this comparison, we determined the ECCP sensitivity to be 97% and the specificity to be 97.5%.

### Validation of the ECCP on serial biopsies from patient DL: mixed histology endometrial adenocarcinoma

To further assess and validate the ECCP against global genome analysis, we analyzed patient DL, for whom we collected serial biopsies (Table 1). To evaluate tumor specific genomic alterations, and to determine if actionable variants could be traced back to the sample from the original diagnosis (DL1), we followed the chronological order of the disease course in this patient. We retrospectively performed a targeted sequencing analysis using the ECCP sequencing of the DL1 endometrial biopsy and DL2 hysterectomy specimens, together with the inguinal lymph node biopsy obtained at recurrence 15.6 month later (DL3).

**Figure 4:**
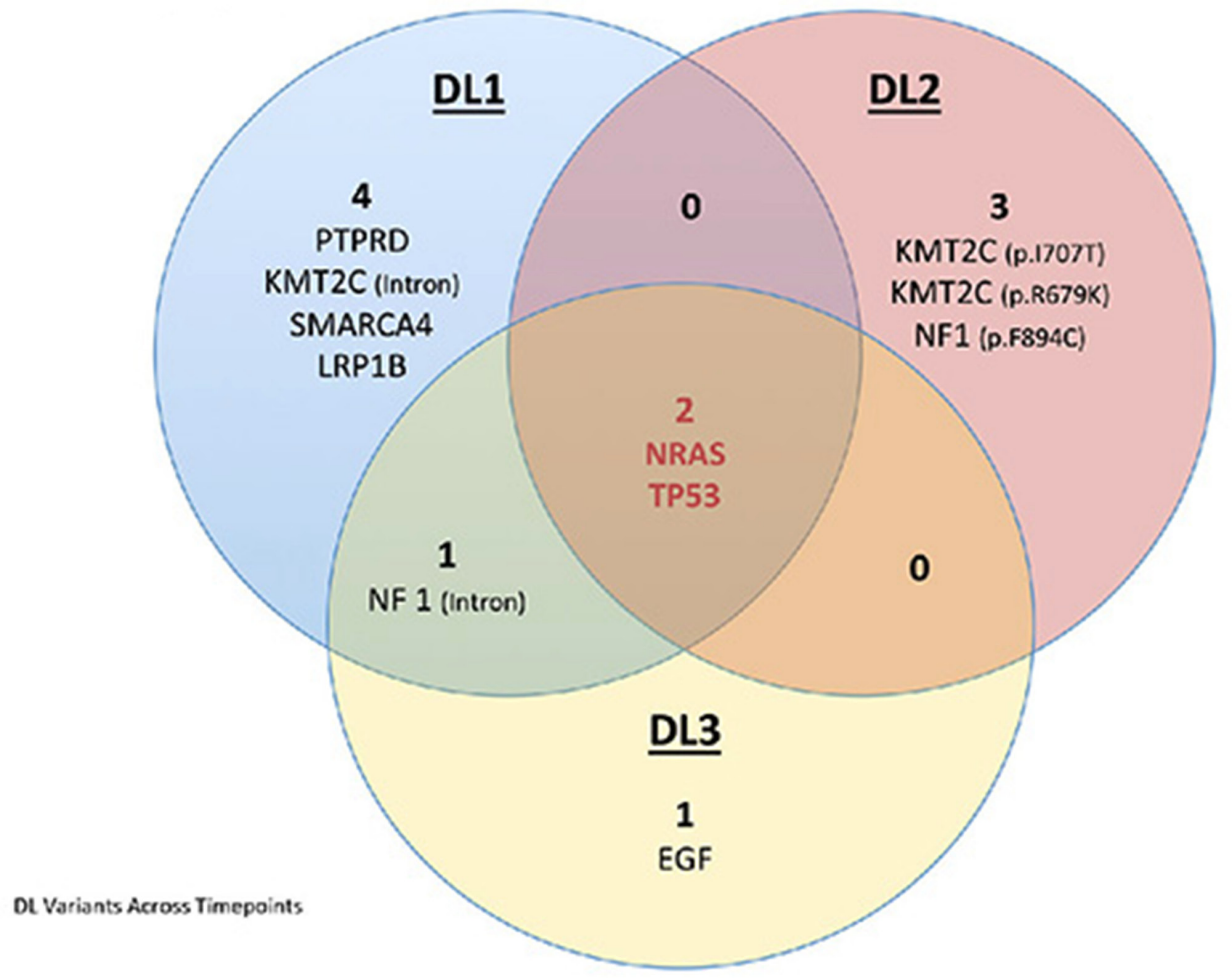
Venn diagram depicting the variants identified in the three DL samples using the targeted ECCP The blue circle depicts the variants identified in DL1, the orange circle depicts the variants identified in DL2, and the yellow circle depicts the variants identified in DL3. The overlapping areas between the circles indicate the common variants, in the center is depicted the variants common to all samples. In red we indicate the driver mutations.

In DL1, we identified 7 variants of interest (Figure 4, Supplementary Table 2). Two variants, likely to be driver mutations, were found in *NRAS* and *TP53*. Both are known to be pathogenic as annotated by ClinVar. In addition, DL1 contained a missense variant of interest mapping to Protein Tyrosine Phosphatase, Receptor Type D (*PTPRD*, p.V1848I). This mutation falls into a 3D cluster mutation hotspot, is predicted to be pathogenic by SIFT and PolyPhen-2, and has been reported in COSMIC in association with colon cancer [19]. An additional missense variant was identified in the LDL Receptor Protein 1B (*LRP1B*), but has no known associations present in COSMIC, TCGA, or ClinVar. The remaining three variants identified in DL1 mapped to non-coding regions of *KMT2C*, SWI/SNF Related, Matrix Associated, Actin Dependent Regulator Of Chromatin, Subfamily A, Member 4 (*SMARCA4*), and Neurofibromin 1 (*NF1*). These variants are of unknown pathogenic potential and have not been previously reported in association with cancer by either TCGA or COSMIC datasets.

In the hysterectomy specimen (DL2), obtained approximately one month after DL1 was biopsied, we identified 5 variants of interest. The two pathogenic variants (*NRAS* and *TP53*) found in DL1 were also identified in DL2. The other three DL2 variants are of unknown pathogenic effect. Two of these are missense variants mapping to *KMT2C* (p.Ile707Thr, p.Arg679Lys). One has been reported in COSMIC in association with lung cancer [20] and the other has been reported by TCGA in association with 1 lung cancer case. A final missense mutation (p.Phe894Cys) in Neurofibromin 1 (*NF1*), a tumor suppressor gene and a negative regulator of *NRAS*, is predicted pathogenic by SIFT/PolyPhen-2 and has been reported in association with one colorectal cancer by TCGA.

Lastly, in our analysis of the recurrent tumor (DL3), we identified 4 variants of interest. The two pathogenic variants mapping to *NRAS* and *TP53* previously identified in DL1 and DL2 were retained in DL3. The other two variants have unknown pathogenic effect. A missense variant in Epidermal Growth Factor *EGF* (p.Pro1096Thr), the upstream regulator of the *NRAS/KRAS* pathway, is predicted pathogenic by SIFT/PolyPhen-2 but has not been reported in association with cancer by the COSMIC or TCGA datasets. The final variant maps to a non-coding region of *NF1*; this same variant was also identified in DL1.

Because the same intronic *NF1* variant was called in both DL1 and DL3, but not DL2, we performed a visual inspection of DL2 in IGV at this locus. It appears that a low read count for this amplicon in DL2 prevented identification of this variant. Additionally, given the low frequency at which some of our variants (*KMT2C* variants, *SMARCA4*, *NF1*, and *EGF*) were identified across our sample set, further validation may be required for those variants.

#### Whole genome sequencing candidate variant results

As a means of validating the DL3 variants identified by the ECCP and to assess if other driver mutations of biological or clinical relevance were missed by the targeted sequencing, we performed WGS of the DL3 specimen. The DL3 analysis was subset into two groups: i) variants with associated COSMIC identifiers, and ii) Cancer Gene Census variants. The COSMIC-associated subset identified 25 variants mapping to coding regions (8 synonymous and 17 missense), including one *NRAS* and one *TP53* variant, and 20 variants in non-coding regions (Supplementary Table 3). In the Cancer Gene Census subset, no high impact variants were identified, though three variants of moderate impact were found in *MLLT4, NRAS*, and *TP53*. The *NRAS* and *TP53* variants identified in the Cancer Gene Census subset are the same variants as noted in the COSMIC-associated subset. Of the variants identified by WGS, the two pathogenic variants in *TP53* and *NRAS* were also identified by the ECCP targeted sequencing. However, the additional *EGF* and *NF1* variants identified in DL3 by the ECCP were not found by WGS. The remainder of the missense variants (COSMIC and Cancer Gene Census) identified by WGS but not the ECCP mapped to genes not included in the ECCP or CCP. Additionally, since both the ECCP and CCP are designed to cover coding regions, none of the 20 non-coding variants were identified using the targeted sequencing approach.

#### RNA sequencing results

To explore the clinical application of targeted sequencing, we next wanted to assess which of the ECCP-identified variants may have contributed to DL's tumorigenesis. To do so, we evaluated, in IGV, the BAM files generated by RNA-Seq of the DL3 sample to screen for potential *NRAS, TP53, EGF, NF1, KMT2C,* and *MAP3K1* transcripts containing the DNA variants identified. Of these, we found that only the *NRAS* and the *TP53* variants were transcribed. We also evaluated these results for potential transcripts of the coding variants with COSMIC entries identified by WGS (see Supplementary Table 3). Of these, we found that in addition to the *NRAS* and *TP53* variants also identified by the ECCP sequencing analysis, only transcripts of Speckle Type BTB/POZ Protein (*SPOP*) and Patatin-Like Phospholipase Domain-Containing Protein 6 (*PNPLA6*) appeared to transcribe the identified DNA variant.

## DISCUSSION

The primary objective of this study was to validate our custom designed targeted sequencing panel with a secondary objective to explore the potential clinical applicability for the identification of actionable variants. To assess the performance of our targeted panel, we performed WES in addition to targeted sequencing using the AmpliSeq Comprehensive Cancer Panel (1.2Mb) and Einstein Custom Cancer Panel (600.2kb). We found that the percentage of on-target reads was higher with the targeted panels than with WES; for example, the percentage of on-target reads for T65 was greater than 96% for the CCP compared with 67.7% for WES. Using targeted sequencing, we were able to identify the driver mutations in both P65 and DL, more economically than possible with WES or WGS, respectively. The smaller size of the targeted panels also allowed for multiplexing of a larger number of samples while still yielding higher coverage. Coverage of T65 using WES was ~83X compared with >700X using the CCP. Thermo Fisher recommends 30X coverage to detect germline mutations using their AmpliSeq technology, but 500X to detect somatic mutations. Depth of coverage is, of course, a function of multiplexing and affordability, but higher coverage depth is required to detect low-frequency somatic variants that have been shown to be associated with resistance to therapy [21].

Initial comparison of WES and targeted sequencing with the ECCP for P65 suggests that the smaller targeted panel failed to map approximately 400 variants. However, scrutiny of the predicted functional impact of the WES-unique variants suggests that their impact is rather modest, as all of the unique variants with pathogenic computational scores were reported in TCGA as putative passenger mutations and none were found to occur in greater than 3.25% of all cancer samples in COSMIC. Eleven predicted-pathogenic variants identified by WES mapped to genes included on at least one of the targeted panels; one *NCOR1* variant was later discarded from the WES results due to questionable quality and high MAF. We surmise that the *NCOR1* variant may be an artifact due to the pseudogenes of *NCOR1* that are found on chromosome 20; this supposition is supported by a few inter-chromosomal rearrangements corresponding to chromosome 20 seen in the visualization of the WES results in this region but not seen in the ECCP results. Of the remaining ten WES variants, nine were also identified by the analyses of the CCP and ECCP, leaving only the *KMT2C* (p.Y987H) variant to be identified by WES but not the targeted panels. In our experience, the correct identification of *KMT2C* variants can be difficult to decipher, due to poor mapping quality, which is likely due to the paralogous sequences known to be part of this gene [22]. Therefore, variants mapping to *KMT2C* should be cautiously considered and validated by Sanger sequencing. Given these considerations, we cannot confirm that this *KMT2C* variant is a true variant of interest. Additionally, the VarScan analysis of the WES failed to identify a *TP53* variant that we suspect to be the driver mutation in P65. Both Ion Reporter CCP and ECCP analyses identified this variant, which was also validated by Sanger Sequencing. Likewise, for DL, the ECCP identified the clinically relevant *TP53* and *NRAS* variants that were identified by WGS. We surmise that the differences in the variant calling may be due, in part, to the greater read depth of the targeted panels (~ 80X coverage for WES compared to ~1000X coverage for the targeted panels), which identified additional variants (*TP53* in P65) that were supported by visual inspection using IGV when compared with WES. These differences in variant calling may also be due to innate variations in the VarScan and Ion Reporter variant calling algorithms, despite consistent parameters. Low concordance between variant callers has been reported, and it has been noted that greater sequencing depth can result in increased agreement between variant callers [23].

When considering the analytical pipelines, VarScan has historically been a gold standard variant caller. However, the Ion AmpliSeq platforms are designed to import sequencing data into Ion Reporter for variant calling and annotation. Ion Reporter is a more streamlined and faster approach, especially for those who are less computationally inclined, making the interface more suitable for clinical settings where a quick turnaround time is of utmost importance. We evaluated the performance of the Ion Reporter variant caller by comparing the variants called by VarScan and Ion Reporter for P65. We found that Ion Reporter identified all of the variants that were identified by VarScan plus 8 additional variants. Importantly, one of these variants was a pathogenic *TP53* variant, which we validated by Sanger sequencing. We also found that Ion Reporter identified the same clinically relevant variants in patient DL as were identified by WGS. From these analyses, we concluded that Ion Reporter adequately mets our needs, performing on par with VarScan as a variant caller.

An exploratory objective of this study was to consider the clinical applicability of targeted next generation DNA sequencing using a mid-size panel larger than that commonly used in clinical settings. To do so, we evaluated the ability of the ECCP to identify actionable and/or driver mutations in DL, a high grade, mixed histology endometrial cancer. In the case of DL, the ECCP identified significantly fewer variants than WGS. However, we identified the pathogenic *NRAS* and *TP53* variant drivers of tumorigenesis. Visual examination of the RNA-Seq data in IGV supports the premise that the *NRAS* and *TP53* variants act as the driver mutations, as the only other variants identified by WGS which are transcribed in DL3 map to *SPOP* and *PNPLA6*. *SPOP* and *PNPLA6* variants both occur at a frequency of less than 1% in endometrial cancers in COSMIC; *PNPLA6* is reported in the TCGA dataset as a passenger mutation in endometrial cancer. The identified *SPOP* variant is noted as a likely oncogenic mutation in 1.2% of the TCGA dataset for endometrial cancer, all corresponding to patients who are living and have all been disease-free for greater than 24 months. This suggests that it is of a low overall impact, which is supported by the variant's Mutation Assessor information in cBioPortal, where it is predicted to have low functional impact, as well as the low overall frequency in COSMIC (0.085%). Though *NRAS* and *TP53* are not currently drugable, targeted therapies are the subject of a significant volume of current research and the number of drugable targets is rapidly expanding. In conclusion, our targeted panel has the ability to identify mutations driving tumorigenesis, as well as actionable variants while minimizing the noise of uninterpretable variants of unknown significance produced by WGS and WES.

## MATERIALS AND METHODS

### Tissue collection and preparation for high throughput sequencing

Written informed consent for genetic studies on tumor, matching peritumoral tissue, and peripheral whole blood was obtained from the patients under IRB protocol 2007-433. The tissues analyzed in this study are summarized in (Table 1).

Breast tumor (T65) and adjacent non-tumor (N65) tissues, sampled from leftover diagnostic surgical tissue, were obtained from P65. Tissue samples were stored in RNAlater at -80°C until use. DNA was extracted from these samples using the QIAamp DNA Mini kit (Qiagen, Hilden, Germany) according to the manufacturer's protocol, in the Molecular Cytogenetic Core Facility at Albert Einstein College of Medicine, Bronx, NY.

Tumor from endometrial biopsy (DL1) and hysterectomy (DL2) specimens, sampled from diagnostic formalin-fixed paraffin-embedded (FFPE) tissue blocks, was obtained from patient DL. Representative H&E stained slides were reviewed by RGK in the department of Pathology at Montefiore Medical Center, who confirmed the original pathologic diagnosis of both specimens (Figure 1). H&E staining was used on matched serial sections to identify areas of tumor that were subsequently grossly microdissected using a scalpel for DNA extraction. Recurrent tumor (DL3) was biopsied at an outside facility and a fresh frozen sample was sent to Albert Einstein College of Medicine for sequencing. Patient DL also provided whole blood (DLWB), which served as a reference to aid in identification of somatic variants. DNA was extracted from DL1 and DL2 specimens using the Qiagen QIAamp DNA FFPE Tissue kit and from DL3 using the Qiagen QIAamp DNA Mini kit, all according to the manufacturer's protocol. Whole blood (~4ml) was processed for genomic DNA isolation using the Puregene Genomic DNA Purification kit (Gentra, MN). The quality of all DNA samples was assessed by the NanoDrop 1000 Spectrophotometer (Thermo Fisher Scientific, Waltham, MA). The Qubit dsDNA HS Assay kit (Thermo Fisher Scientific, Waltham, MA) was used to determine the concentration of each DNA sample. Samples were subsequently run on a 1% Agarose-TBE Blend gel to evaluate the DNA integrity and identify impurities.

### High throughput sequencing

#### Illumina sequencing

##### Whole genome sequencing (WGS)

Samples DL3 and DLWB were analyzed by whole genome sequencing (WGS). One microgram of DNA was used to construct standard 2 × 100 bp libraries using the Illumina TruSeq Library Preparation kit with the standard protocol (Illumina, San Diego, CA) after fragmentation on the Covaris (Covaris, Woburn, MA) as previously described [10]. Libraries were sequenced at the New York Genome Center (New York, NY) on the Illumina HiSeq 2500, running one sample per lane.

##### Whole exome sequencing (WES)

Samples N65 and T65 were analyzed by whole exome sequencing (WES). Sequencing libraries were constructed starting from 1 μg of input genomic DNA using the Illumina TruSeq Library Preparation kit, according to the manufacturer's protocol. The whole exome target regions were then captured using the NimbleGen v3 (64 Mb) Whole Exome Enrichment kit (Roche/NimbleGen, Madison, WI), according to the manufacturer's protocol. Both sequencing libraries were then sequenced on a single lane of the HiSeq 2000 (Illumina, San Diego, CA) to generate 2 × 100bp paired end sequencing. WES was performed using Axeq Technologies’ Illumina sequencers (Macrogen Inc, South Korea).

##### RNA Sequencing (RNA-Seq)

Sample DL3 was used for RNA-Seq analysis. A sequencing library for the Illumina 2500 platform was created from the polyadenylated fraction of RNA. mRNA was isolated with Dyna1 oligo-dT beads (Thermo Fisher Scientific, Waltham, MA) from 10 μg of total RNA. The mRNA was randomly fragmented using the RNA Fragmentation kit from Ambion. First-strand cDNA synthesis was performed using random primers and SuperScriptII Reverse-Transcriptase (Thermo Fisher Scientific, Waltham, MA). This was followed by second-strand cDNA synthesis using DNA Polymerase I and RNase H (Thermo Fisher Scientific, Waltham, MA). The Illumina adaptor was ligated to the ends of the double-stranded cDNA fragments and a 200 bp size-selection of the final product was performed by gel-excision, following the Illumina-recommended protocol. 200 bp adapter-ligated cDNA template fragments were enriched by PCR to create the final library. The DL3 RNA-Seq library was sequenced at the New York Genome Center in one lane on the Illumina HiSeq 2000 Sequencing System, using 2 × 100bp reads.

#### Ion AmpliSeq targeted sequencing

##### Comprehensive Cancer Panel (CCP)

Samples N65 and T65 were sequenced using the CCP. The Ion AmpliSeq Library Kit 2.0 and the Ion AmpliSeq Comprehensive Cancer Panel (Thermo Fisher Scientific, Waltham, MA) were used to sequence 409 cancer-associated genes (complete gene list is provided in Supplementary Appendix 3). For each sample, a PCR reaction was set up according to the manufacturer's protocol using 40 ng of genomic DNA, four separate primer pools, and 13 amplification cycles. Following amplification, each sample was treated with FuPa reagent and ligated to a uniquely barcoded adapter to enable sample multiplexing. Libraries were then purified using 1.5X Agencourt AMPure XP (Beckman Coulter Inc, Brea, CA) kit and eluted in 50 μl of low TE. Amplification products from each primer pool were quantified individually using the Ion Library Quantitation kit (Thermo Fisher Scientific, Waltham, MA) and then pooled together. Template preparation was performed using the Ion OneTouch 2 system and the Ion PI Template OT2 200 kit v2 (Thermo Fisher Scientific, Waltham, MA), according to the manufacturer's protocol. Both libraries were sequenced on the Ion Proton sequencer using the Ion PI chip and the Ion PI Sequencing 200 kit v2 (Thermo Fisher Scientific, Waltham, MA) to generate 200bp single ended sequencing.

##### Einstein Custom Cancer Panel (ECCP)

N65, T65, and all DL samples were sequenced using the ECCP. Targeted next generation sequencing of 156 (Supplementary Appendix 1) known and suspected cancer-related genes was performed using the custom-designed ECCP. Using 10 ng of DNA in each of two primer pools, target genes were amplified in 10 μL reactions using the Ion AmpliSeq Library Preparation kit 2.0 (Thermo Fisher Scientific, Waltham, MA) and the Ion AmpliSeq Einstein Custom Cancer Panel, following the manufacturer's protocol to produce DNA libraries. For the initial PCR amplification step, 17 and 21 PCR cycles were used for fresh frozen and FFPE tissue, respectively, per manufacturer's recommendations based upon the number of amplicons per primer pool and tissue type. Following amplification, the samples were treated with FuPa reagent, provided in the Ion AmpliSeq Library Preparation kit 2.0 (Thermo Fisher Scientific, Waltham, MA). A uniquely barcoded IT Xpress adapter was then ligated to each sample, thereby allowing multiplex sequencing of the samples. The ligated products were purified using 1.5X Agencourt AMPure XP (Beckman Coulter Inc, Brea, USA) kit. The purified library was eluted in 50 μL low TE (included with Ion AmpliSeq Library Preparation kit 2.0). The purified libraries were diluted 1:100 and quantified using the Applied Biosystems StepOne Plus real-time qPCR system and either Applied Biosystems’ Ion Library Quantitation kit or KAPA Biosystems’ Ion AmpliSeq Library Quantitation kit. Quantified, purified libraries were diluted to 100 pM and pooled together for sequencing. Only libraries quantified using the same quantification kit were sequenced simultaneously. The Ion OneTouch 2 system (Thermo Fisher Scientific, Waltham, MA) was used to amplify the library fragments onto Ion Sphere Particles (ISPs) provided with the Ion PI Template OT2 200 kit v2 (Thermo Fisher Scientific, Waltham, MA). The Ion Sphere Quality Control kit with the Qubit 2.0 fluorometer (Thermo Fisher Scientific, Waltham, MA) was used to assess the template efficiency of ISPs, ensuring that the percent of templated ISPs was between 10 and 30%. Sequencing was performed on the Ion Proton platform (Thermo Fisher Scientific, Waltham, MA), using PI sequencing chip (Thermo Fisher Scientific, Waltham, MA) and Ion PI Sequencing 200 Kit v3 (Thermo Fisher Scientific, Waltham, MA), according to manufacturer's guidelines.

##### High throughput sequencing analysis

Data analysis was performed using different bio-informatics tools, specific to the sequencing platform and the capture approach used.

***WGS:*** We used a combination of analytical tools to map single nucleotide variants in sample DL3: MuTect under High-Confidence mode with default parameter settings [24], which detects somatic point mutations using pre-processing aligned reads separately in tumor and normal samples; Strelka [25], which reports the most likely genotype for tumor and normal samples based on a Bayesian probability model); and Virmid [26].

***WES:*** WES raw data was processed as previously described [10]. Variant calling and annotation were performed with SAMTools (http://samtools.sourceforge.net 0.1.18) and VarScanV211 (http://dkoboldt.github.io/varscan/somatic-calling.html) [27], and the output was annotated for further investigation.

***RNA-Seq:*** All statistical methods, and data analysis were conducted using the R statistical software [28] as we previously described [29].

### Ion AmpliSeq CCP and ECCP

After sequencing, reads were analyzed using the Ion Torrent Suite's Torrent Variant Caller (TVC), using v4.0 for P65 and v4.2 for DL, under low stringency parameters, based on the company's recommendation for custom panels. (Thermo Fisher Scientific, Waltham, MA). The reads from each sample were aligned to the human reference genome from NCBI (hg19-Genome Reference Consortium GRCh37). The generated BAM files were also imported into the Ion Reporter Software, versions 4.0 and 4.4, (Thermo Fisher Scientific, Waltham, MA) for variant calling and annotation. Variant calls for each tumor sample were analyzed against those identified in the matching normal sample using Ion Reporter Software, v4.0 for P65 and v4.4 for DL, to identify tumor specific variants. The DL samples were later re-analyzed under a newer version (v5.0); variants appearing in both variant callers were considered to be stronger candidates as true variants, but significant differences in the variant calling were not observed.

Variants were filtered by p-value (removing p-value > 0.05), minor allele frequency (removing variants with MAF > 1%), and variant functional consequence (removing synonymous variants). Tools of population genetics (Sorting Intolerant from Tolerant, SIFT; and Polymorphism Phenotyping v2, PolyPhen-2) were used to predict pathogenicity of amino acid changes of nonsynonymous SNVs. SIFT scores < 0.05 imply deleterious function, and PolyPhen-2 scores range 0 to 1, with higher scores implying deleterious function. Variants predicted to be benign by both PolyPhen-2 < 0.5 and SIFT > 0.05 were filtered out. To assess the quality of the variant call, all variants of interest identified using the bioinformatics pipeline were visually inspected at the sequence level using the Broad Institute's Integrative Genomics Viewer (IGV) [30].

We first annotated filtered variants of interest by interrogating databases that report previously identified pathogenic variants (e.g., ClinVar). Variants with unreported pathogenicity profile were annotated according to basic functional information (such as gene, variant type gene region, variant function), as well as SIFT/PolyPhen-2 predicted pathogenicity. Finally, The Cancer Genome Atlas (TCGA) and Catalog of Somatic Mutations in Cancer (COSMIC) databases were referenced to assess the frequency of variants of interest in previously reported tumors, and literature searches were performed to identify whether the variant occurred within an exon frequently associated with disease. Based on annotative results, pathogenic variants of interest and candidate genes were identified.

### Validation

A *TP53* variant, previously reported as pathogenic in ClinVar in relation to Li-Fraumeni syndrome and/or hereditary cancer pre-disposing syndrome, was found in P65 and validated using Sanger sequencing. Primers were designed using primer3 v0.4.0 (bioinfo.ut.ee/primer3-0.4.0/). SNPs in the primer-binding site were ruled out using the NGRL SNPCheck database (https://ngrl.manchester.ac.uk/SNPCheckV3/snpcheck) prior to ordering. PCR amplification was performed using the FASTstart High Fidelity PCR system (Roche, Madison, WI) at 59°C annealing temperature. Amplified PCR products were then purified using the AMPure Purification System (Beckman Coulter, Indianapolis, IN). The purified products were sequenced on the Applied Biosystems 3730 sequencer (Genomics Core at Einstein, NY). The Sequencer v4.0.1 software (Gene Codes, Ann Arbor, MI) was used to analyze sequencing files.

## CONCLUSIONS

Based on our extensive comparisons, the sequencing technologies available to us, and the focus on cancer-related genes, we determined that the Ion Proton sequencing platform coupled with the AmpliSeq capture technology offers a sensitive and streamlined approach for the analysis of the cancer genome with a short timeframe, and eliminates much of the background noise generated by WES, which is imperative to the eventual translation of sequencing technologies to the clinical setting. Furthermore, the ECCP accurately assessed the majority of driver cancer mutations for the samples analyzed here. Taken together with the lower costs of library preparation and sequencing and the ability to use less input genomic DNA when compared to the larger CCP or WES/WGS, we feel the choice to use our custom panel is validated.

## SUPPLEMENTARY MATERIALS FIGURES AND TABLES














