# Supplementary material for: Development and validation of a targeted next generation DNA sequencing panel outperforming whole exome sequencing for the identification of clinically relevant genetic variants
Source: Oncotarget. 2017 Oct 26;8(60):102033–45. doi: 10.18632/oncotarget.22116 (PMC5731933; doi:10.18632/oncotarget.22116)
Supplement: Supplementary file 1 [file oncotarget-08-102033-s001.pdf]

# Development and validation of a targeted next generation DNA sequencing panel outperforming whole exome sequencing for the identification of clinically relevant genetic variants

## SUPPLEMENTARY MATERIALS

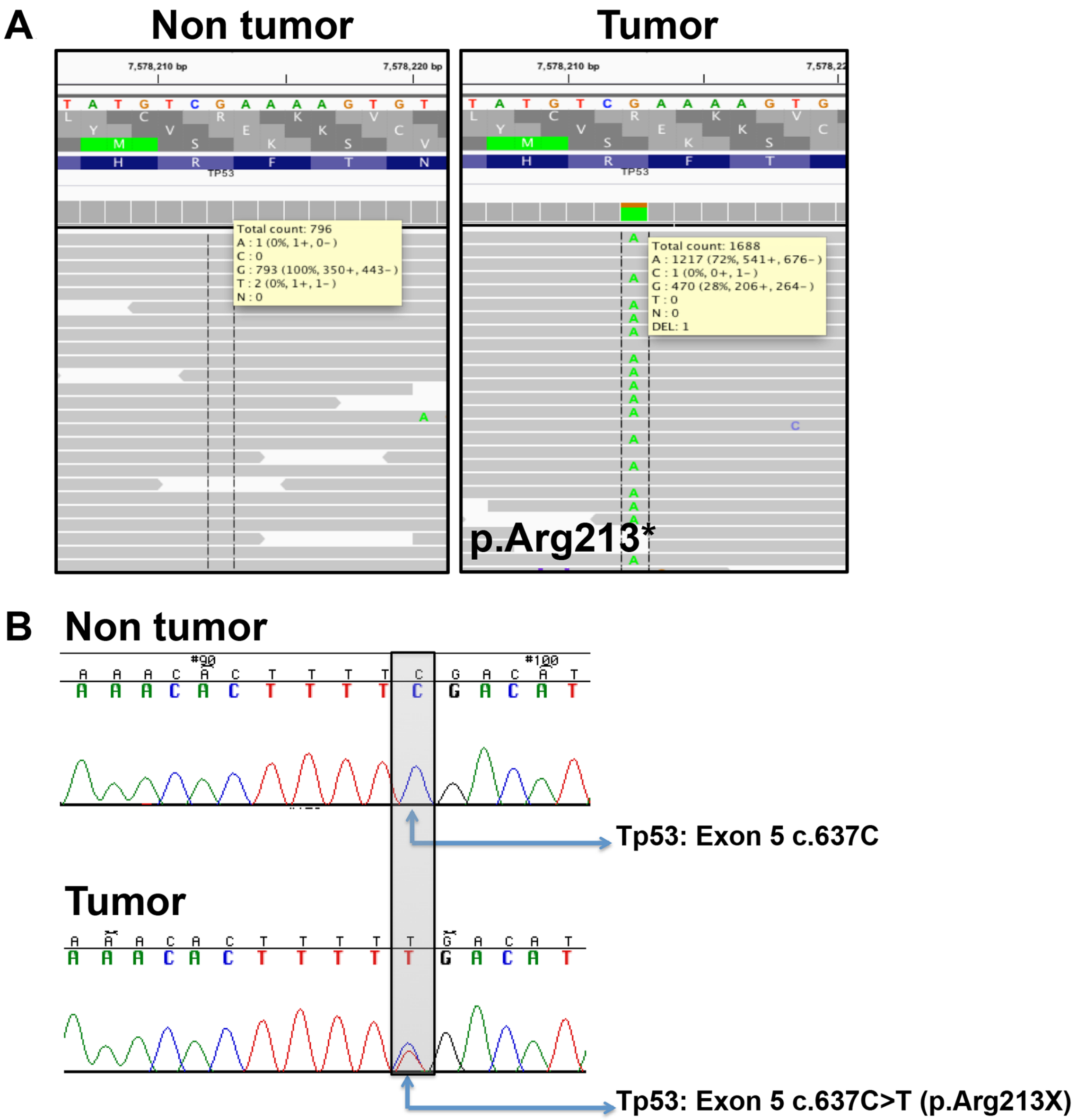


**Supplementary Figure 1: Sanger sequencing validation of the TP53 variant identified in P65. (A)** Screenshot of the sequences reads aligned to the reference genome using the Integrative Genomics Viewer (IGV). Left panel non-tumor tissue, right panel tumor tissue. **(B)** Sanger sequencing chromatograms of the TP53 mutation.

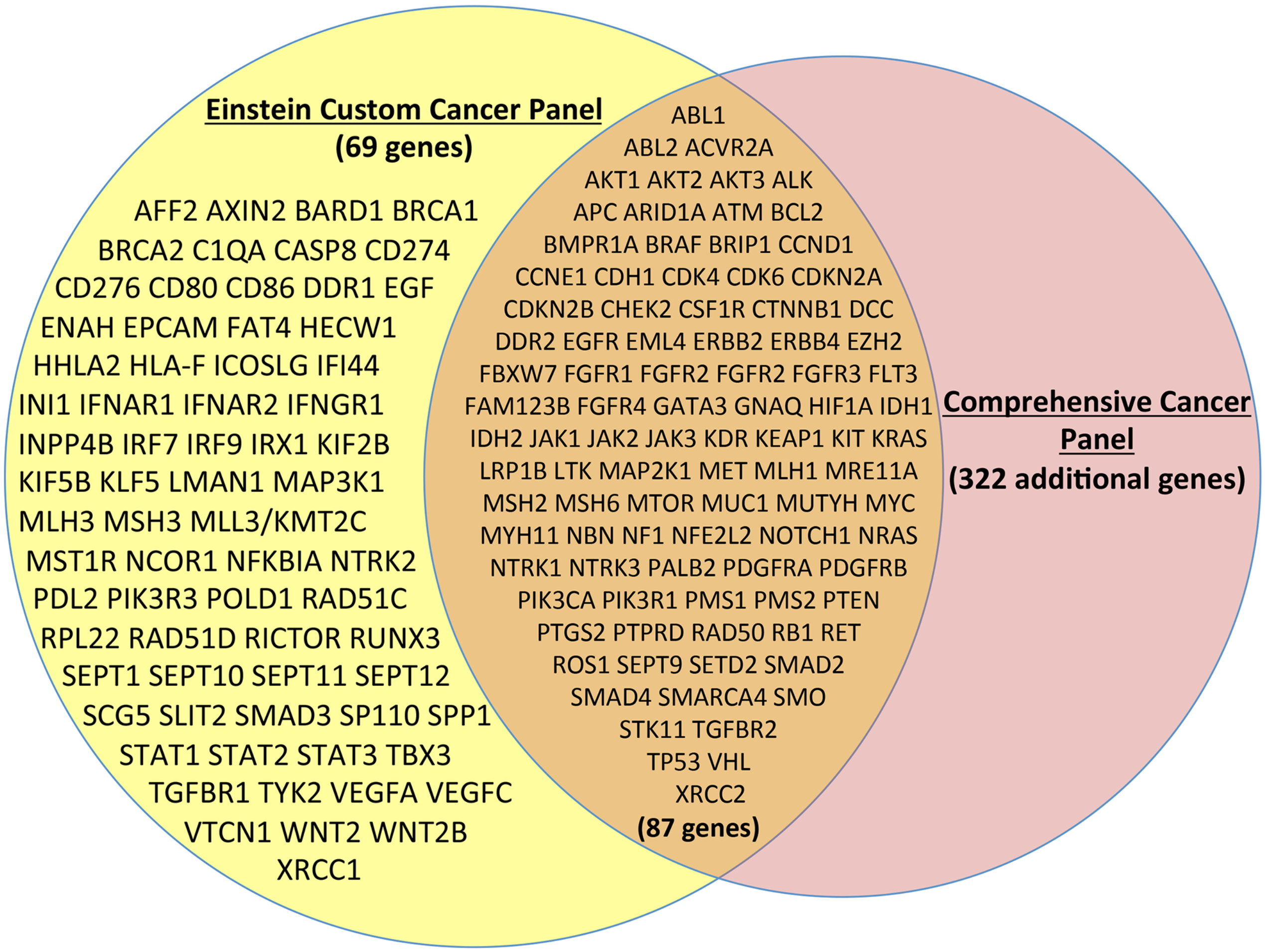


**Supplementary Figure 2: Venn diagram listing the overlap of genes between the ECCP and the CCP.**

**Supplementary Table 1: Comparison of Results of WES, CCP, and ECCP Analyses for P65.**

**See Supplementary File 1**

**Supplementary Table 2: DL Variants Identified by ECCP.**

**See Supplementary File 2**

**Supplementary Table 3: DL2 Whole Genome Sequencing Variant Results.**

**See Supplementary File 3**

**Appendix 1: List of genes included on the Einstein Custom Cancer Panel.**

**See Supplementary Appendix 1**

**Appendix 2: List of variants identified by WES for P65.**

**See Supplementary Appendix 2**

**Appendix 3: List of genes included on the Comprehensive Cancer Panel.**

**See Supplementary Appendix 3**
