# Supplementary material for: Development and validation of a targeted next generation DNA sequencing panel outperforming whole exome sequencing for the identification of clinically relevant genetic variants
Source: Oncotarget. 2017 Oct 26;8(60):102033–45. doi: 10.18632/oncotarget.22116 (PMC5731933; doi:10.18632/oncotarget.22116)
Supplement: Supplementary file 5 [file oncotarget-08-102033-s005.docx]

| Locus | Gene | Transcript | Coding | Amino Acid Change | Variant Effect | dbSNP | # of Analyses Called By | In WES | Reason Not Called /  Other Info | In CCP | Reason Not Called /  Other Info | In ECCP | Reason Not Called /  Other Info | Notes |
| --- | --- | --- | --- | --- | --- | --- | --- | --- | --- | --- | --- | --- | --- | --- |
| chr1:45798329 | MUTYH | NM_001128425.1 | c.607C>T | p.R203C | missense |  | 2 | No |  | Yes |  | Yes |  |  |
| chr2:216274456 | FN1 | NM_212482.1 | c.2129C>T | p.T710I | missense | rs79328281 | 2 | Yes |  | Yes |  | No | Not in Panel |  |
| chr3:14220044 | XPC | NM_014463.2 | c.25G>T | p.G9W | missense |  | 2* | No* | PolyPhen-2 Score | Yes |  | No | Not in Panel |  |
| chr3:49928691 | MST1R | NM_002447.2 | c.3583A>G | p.S1195G | missense | rs7433231 | 1 | No |  | No | Not in Panel | Yes |  |  |
| chr4:126373789 | FAT4 | NM_024582.4 | c.11618G>A | p.S3873N | missense | rs12650153 | 1 | No |  | No | Not in Panel | Yes |  | Later filtered out - ClinVar |
| chr5:112178073 | **APC** | **NM_000038.5** | **c.6782C>T** | **p.P2261L** | **missense** | **rs376494248** | **3** | **Yes** |  | **Yes** |  | **Yes** |  |  |
| chr6:160485549 | IGF2R | NM_000876.2 | c.4003C>T | p.R1335C | missense | rs144472377 | 2 | Yes |  | Yes |  | No | Not in Panel |  |
| chr7:142566782 | EPHB6 | NM_004445.4 | c.2339C>T | p.A780V | missense |  | 2 | Yes |  | Yes |  | No | Not in Panel |  |
| chr11:118374739 | KMT2A/MLL | NM_001197104.1 | c.8132A>C | p.Q2711P | missense |  | 2 | Yes |  | Yes |  | No | Not in Panel |  |
| chr12:43822322 | ADAMTS20 | NM_025003.3 | c.3667T>C | p.C1223R | missense | rs151010911 | 2 | Yes |  | Yes |  | No | Not in Panel |  |
| chr13:103527849 | ERCC5 | NM_001204425.1 | c.4519G>C | p.G1507R | missense | rs9514066 | 1 | No |  | Yes |  | No | Not in Panel |  |
| chr13:32911452 | BRCA2 | NM_000059.3 | c.2960A>T | p.N987I | missense | rs2227944 | 1 | No |  | No | Not in Panel | Yes |  | Later filtered out - ClinVar |
| chr15:43569097 | TGM7 | NM_052955.2 | c.1936G>A | p.E646L | missense | rs146833656 | 2* | No* | PolyPhen-2 Score | Yes | Low read count | No | Not in Panel |  |
| chr16:23649446 | PALB2 | NM_024675.3 | c.53A>G | p.L18R | missense | rs138789658 | 2 | No |  | Yes |  | Yes |  | Later filtered out - ClinVar |
| chr7:151945007 | KMT2C/MLL3 | NM_170606.2 | c.2512G>A | p.G838S | missense | rs2479172 | 2 | Yes |  | No | Poor mapping  quality | Yes | Poor mapping  quality | MAF >1% |
| chr7:151927025 | KMT2C/MLL3 | NM_170606 | c.2959T>C | p.Y987H | missense | rs77735469 | 1 | Yes |  | No | Insufficient depth;  Poor mapping  quality | No | Low Frequency;  Poor mapping  quality |  |
| chr17:7578212 | TP53 | NM_000546.5 | c.637C>T | p.R213* | nonsense | rs397516436 | 2 | No | VARSCAN Analysis | Yes |  | Yes |  | Validated Variant |
| chr17:16068377 | NCOR1 | NM_001190440 | c.534G>C | p.K178N | nonsense | rs200020868 | 1 | Yes |  | No | Not in Panel | No | Potentially  WES error | MAF >1% |
| chr20:40733305 | PTPRT | NM_133170.3 | c.3501C>A | p.N1167L | missense | rs201162919 | 2 | Yes |  | Yes |  | No | Not in Panel |  |
| chr21:46309312 | ITGB2 | NM_000211.3 | c.1756C>T | p.R586W | missense | rs5030672 | 2 | Yes |  | Yes |  | No | Not in Panel |  |
| chr21:46314907 | ITGB2 | NM_000211.3 | c.1062A>T | p.Q354H | missense | rs235330 | 1 | No |  | Yes |  | No | Not in Panel |  |

**Supplementary Table 1: Comparison of Results of WES, CCP, and ECCP Analyses for P65**
