# Supplementary material for: Development and validation of a targeted next generation DNA sequencing panel outperforming whole exome sequencing for the identification of clinically relevant genetic variants
Source: Oncotarget. 2017 Oct 26;8(60):102033–45. doi: 10.18632/oncotarget.22116 (PMC5731933; doi:10.18632/oncotarget.22116)
Supplement: Supplementary file 6 [file oncotarget-08-102033-s006.docx]

| Sample | Locus (mapping on Hg19) | Type | Gene | aa substitution | | SIFT/ PolyPhen | | TCGA (frequency of aa mutation) | | TCGA uterine (frequency of aa mutation) | | COSMIC | | ClinVar | Alt Allele Frequency in Sample | | Pathogenicity classification | |  |
| --- | --- | --- | --- | --- | --- | --- | --- | --- | --- | --- | --- | --- | --- | --- | --- | --- | --- | --- | --- |
| DL1  DL2  DL3 | chr1:115256529 | SNV | NRAS | Q61R | No Info | | 52.1% | | 44.4% | | > 1000 p.Gln62 missense variants | | Pathogenic | | | 63.46%  49.00%  84.88% | | Pathogenic | |
| DL1  DL2  DL3 | chr17:7577120 | SNV | TP53 | R273H | No Info | | 3.7% | | 12.1% | | >1000 p.Arg missense variants | | Pathogenic | | | 50.60%  36.04%  74.47% | | Pathogenic | |
| DL1 | chr9:8319959 | SNV | PTPRD | V1848I | Damaging | | 0% | | 0% | | COSM4631406 | | No Entry | | | 18.40% | | VUS - Interest | |
| DL1 | chr2:141128335 | SNV | LRP1B | L3651 | No Info | | 0% | | 0% | | No Entry | | No Entry | | | 39.02% | | VUS | |
| DL1 | chr7:151919649 | SNV | KMT2C | Intronic | No Info | | NA | | NA | | No Entry | | No Entry | | | 8.65% | | VUS | |
| DL1 | chr19:11102073 | SNV | SMARCA4 | Intronic | No Info | | NA | | NA | | No Entry | | No Entry | | | 9.26% | | VUS | |
| DL1  DL3 | chr17:29684279 | SNV | NF1 | Intronic | No Info | | NA | | NA | | No Entry | | Uncertain Significance | | | 52.16%  79.12% | | VUS | |
| DL2 | chr7:151945399 | SNV | KMT2C | I707T | No Info | | 0% | | 0% | | COSM97020 | | No Entry | | | 5.63% | | VUS - Interest | |
| DL2 | chr7:151945483 | SNV | KMT2C | R679K | No Info | | 0.06% | | 0% | | No Entry | | No Entry | | | 5.87% | | VUS | |
| DL2 | chr17:29556314 | SNV | NF1 | F894C | Damaging | | 0% | | 0% | | No Entry | | No Entry | | | 4.80% | | VUS | |
| DL3 | chr4:110925773 | SNV | EGF | P1096T | Damaging | | 0% | | 0% | | No Entry | | No Entry | | | 7.88% | | VUS | |

**Supplementary Table 2: DL Variants Identified by ECCP**
