# Supplementary material for: Development and validation of a targeted next generation DNA sequencing panel outperforming whole exome sequencing for the identification of clinically relevant genetic variants
Source: Oncotarget. 2017 Oct 26;8(60):102033–45. doi: 10.18632/oncotarget.22116 (PMC5731933; doi:10.18632/oncotarget.22116)
Supplement: Supplementary file 7 [file oncotarget-08-102033-s007.docx]

**Supplementary Table 3: Whole Genome Sequencing Variant Results**

| **Gene** | **Database** | **COSMIC ID/**  **CGS Variant Effect** | **Locus** | **CDS (AA Change)** | **Gene in**  **ECCP** | **Gene in**  **CCP** |
| --- | --- | --- | --- | --- | --- | --- |
| **Coding** | | | | | | |
| TMEM14B | COSMIC | COSM226573 | chr6:10756728 | c.322C>T (p.R108C) | No | No |
| ZVF181 | COSMIC | COSM565483 | chr19:35232200 | c.722T>G (p.V241G) | No | No |
| PCDHA8 | COSMIC | COSM225565 | chr5:140221195 | c.289G>C (p.G97R) | No | No |
| SPOP | COSMIC | COSM980818 | chr17:47699360 | c.148G>A (p.E50K) | No | No |
| RBM23 | COSMIC | COSM1240025 | chr14:23371268 | c.1167T>C (p.A389A) | No | No |
| DHX34 | COSMIC | COSM474961 | chr19:47870384 | c.1740A>G (p.L580L) | No | No |
| POLR2A | COSMIC | COSM707187 | chr17:7386217 | c.917T>C (p.V305A) | No | No |
| IFNA4 | COSMIC | COSM403873 | chr9:21187444 | c.87C>T (p.T29T) | No | No |
| MUC21 | COSMIC | COSM222094 | chr6:30954572 | c.620C>T (p.A207V) | No | No |
| FRG2B | COSMIC | COSM3666722 | chr10:135438806 | c.634C>A (p.R212R) | No | No |
| MEF2A | COSMIC | COSM321647 | chr15:100252744 | c.1262C>A (p.P421Q) | No | No |
| PTPRT | COSMIC | COSM246890 | chr20:40790169 | c.2571C>T (p.R857R) | No | **Yes** |
| XCL2 | COSMIC | COSM1579705 | chr1:168511321 | c.86G>A (p.R29K) | No | No |
| TBP | COSMIC | COSM1442363 | chr6:170871058 | c.234G>A (p.Q78Q) | No | No |
| FLJ22184 | COSMIC | COSM1613086 | chr19:7935863 | c.2267C>A (p.P756H) | No | No |
| AHNAK2 | COSMIC | COSM1283330 | chr14:105412347 | c.9441G>A (p.K3147K) | No | No |
| KRTAP1-1 | COSMIC | COSM979031 | chr17:39197248 | c.402C>T (p.C134C) | No | No |
| TPSD1 | COSMIC | COSM226769 | chr16:1306817 | c.274G>A (p.A92T) | No | No |
| PER3 | COSMIC | COSM1134947 | chr1:7890053 | c.3019G>A (p.A1007T) | No | No |
| PNPLA6 | COSMIC | COSM1647009 | chr19:7625941 | c.3888G>C (p.E1296D) | No | No |
| MUC4 | COSMIC | COSM479915 | chr3:195510146 | c.9305C>G (p.L2769V) | No | No |
| POLR2A | COSMIC | COSM231364 | chr17:7386234 | c.931G>A (p.V311M) | No | No |
| CASP1 | COSMIC | COSM226694 | chr11:104905100 | c.109A>C (p.K37Q) | No | No |
| **NRAS*** | **COSMIC/**  **Cancer Gene Census** | **COSM584/**  **Non-Synonymous Coding** | chr1:115256529 | c.182A>G (p.Q61R) | **Yes** | **Yes** |
| **TP53*** | **COSMIC/**  **Cancer Gene Census** | **COSM3356963/**  **Non-Synonymous Coding** | chr17:7577120 | c.818G>A (p.R273H) | **Yes** | **Yes** |
| MLLT4 | Cancer Gene Census | Non-Synonymous Coding | chr6:168273054 | c.572A>T (p.D191V) | No | No |
| **Non-Coding** | | | | | | |
| NA | COSMIC | COSN208029 | | | | |
| NA | COSMIC | COSN147643 | | | | |
| NA | COSMIC | COSN150592 | | | | |
| NA | COSMIC | COSN148021 | | | | |
| NA | COSMIC | COSN391740 | | | | |
| KIR2DS4 | COSMIC | COSN168937 | | | | |
| NA | COSMIC | COSN207020 | | | | |
| NA | COSMIC | COSN155495 | | | | |
| NA | COSMIC | COSN161250 | | | | |
| NA | COSMIC | COSN394176 | | | | |
| NA | COSMIC | COSN399162 | | | | |
| CHRNA7 | COSMIC | COSN199857 | | | | |
| NA | COSMIC | COSN147152 | | | | |
| NA | COSMIC | COSN138093 | | | | |
| TSC1 | COSMIC | COSN166004 | | | | |
| NA | COSMIC | COSN392600 | | | | |
| NA | COSMIC | COSN394871 | | | | |
| NA | COSMIC | COSN153768 | | | | |
| ZNF285 | COSMIC | COSN394654 | | | | |
| NA | COSMIC | COSN391372 | | | | |

* Identified by targeted sequencing
